# The effects of Omarigliptin on promoting osteoblastic differentiation

**DOI:** 10.1080/21655979.2021.1999366

**Published:** 2021-12-19

**Authors:** Fake Liao, Xiunian Hu, Rijiang Chen

**Affiliations:** Department of Orthopedics, Longyan First Affiliated Hospital of Fujian Medical University, Longyan City, Fujian Province, China

**Keywords:** Omarigliptin, Osteoporosis, osteoblastic differentiation, RUNX2

## Abstract

Osteoporosis significantly impacts the normal life of the elderly and is reported to be closely related to dysfunction of osteoblastic differentiation. Runt-related transcription factor-2 (Runx2) is a critical transcriptional factor involved in the regulation of osteoblast differentiation. Omarigliptin is a novel dipeptidyl peptidase-4 (DDP-4) inhibitor and this study proposes to probe into its possible therapeutic function against Osteoporosis by investigating its impacts on osteoblastic differentiation. Osteogenic medium was used to induce osteoblastic differentiation in MC3T3‑E1 cells, and was verified by the increased alkaline phosphatase (ALP) activity, enhanced mineralization, and promoted expression level of osteoblastic differentiation-related factors, including bone morphogenetic protein-2 (BMP-2), ALP, osteocalcin (Ocn), collagen type I alpha 1 (Col1a1), Collagen Type I alpha 2 (Col1a2), Runx2, osterix (Sp7), fibroblast growth factor receptor 2 (Fgfr2), and fibroblast growth factor receptor 3 (Fgfr3), accompanied by the activation of the p38 and Akt pathways. After treatment with Omarigliptin, the ALP activity and mineralization were further promoted, accompanied by the further upregulation of osteoblastic differentiation-related factors, and activation of the p38 and Akt pathways. Lastly, Omarigliptin-induced osteoblastic differentiation, promoted ALP activity, and increased expression levels of Sp7, Fgfr2, Fgfr3, BMP-2, Ocn, ALP, Col1a1, and Col1a2, in the osteogenic medium- cultured MC3T3‑E1 cells were dramatically abolished by the knockdown of Runx2. Taken together, our data reveal that Omarigliptin promoted osteoblastic differentiation by regulating Runx2.

## Introduction

Osteoporosis is an orthopedic disease mainly observed in the elderly and it affects females more than males [1]. Osteoporosis not only induces significant injuries both mentally and physiologically but is also an important inducer for other diseases, such as bedsores, which further elevates the mortality of patients. In addition, significant financial burdens are brought to society and families by Osteoporosis [2]. The main pathological factor that induces the development of Osteoporosis is that bone resorption is faster than bone formation. The balance between bone resorption and formation can be regulated by multiple cytokines released by osteoblasts and osteoclasts [3]. In the bone resorption area, new bone tissue is formed by osteoblasts through absorbing fragmentary bone tissues destroyed by osteoclasts [4]. Therefore, osteoblastic differentiation and function play important roles in the development and processing of osteoporosis [5]. Several factors have been reported to regulate the processing of osteoblastic differentiation. Alkaline phosphatase (ALP) is an important tissue nonspecific enzyme that promotes the progression of osteoblastic differentiation [6], and is regarded as a mature biomarker for the diagnosis of Osteoporosis [7]. Runx2 is a transcriptional factor containing the runt domain gene family and mediates the proliferation and differentiation of osteoblasts [8,9]. Bone morphogenetic proteins (BMPs) have been claimed to activate ALP at a certain stage of osteoblastic differentiation and induce osteoblastic differentiation from mesenchymal stem cells (MSCs) by triggering the cell mineralization and activating osteoblast-specific transcription factors [10,11]. In C3H10T1/2 cells, BMP-2 regulates osteoblastic differentiation by mediating several enzymes [12].Sp7, a crucial transcription factor for osteoblast differentiation, is found to regulate the expression levels of bone matrix genes, such as *Spp1, Ibsp*, and *Bglap2* [13]. Apart from these factors, several pathways have been claimed to be involved in osteoblast differentiation. The P38 mitogen-activated protein kinase (p38MAPK) pathway is reported to regulate the expression level of BMP-2 and the processing of osteoblast differentiation induced by regular methods [14]. In addition, after the activation of the PI3K/Akt pathway, induced by growth factors, the differentiation of osteoblasts is facilitated and their apoptosis is suppressed by effective downstream proteins of the PI3K/Akt pathway, such as mTORC1 and S6K1 [15]. It is important for the treatment of Osteoporosis to deeply investigate the pathological mechanism of osteoblastic differentiation.

Omarigliptin is an effective and highly selective inhibitor of DPP-4, with much higher inhibitory activity against DPP-4 than Sitagliptin [16]. With a relatively long half-life (63 h) and effective hypoglycemic activity, Omarigliptin is clinically utilized for the treatment of type II diabetes. Interestingly, a wide range of pharmacological functions of Omarigliptin have been reported before. However, little is known about the roles of Omarigliptin in bone metabolism disease. The present study aims to explore the regulatory effect of Omarigliptin on osteoblastic differentiation to investigate its potential in treating Osteoporosis.

## Materials and methods

### Cell culture and treatments

The rodent pre-osteoblastic cell line MC3T3‑E1 cells were obtained from the American Type Culture Collection (ATCC) (Virginia, USA) and cultured in α-Minimum Essential Medium (α-MEM) (Grand Island, USA) containing 10% FBS at 5% CO_2_ and 37°C. Osteogenic medium (Sigma, Missouri, USA) was utilized to culture MC3T3‑E1 cells for 14 days for osteogenic differentiation, with the medium being replaced every 2 days. Omarigliptin was purchased from Invivochem (#V2771), USA.

### Lenti-viral transduction

To knockdown the expression level of Runx2 in MC3T3‑E1 cells, the lentiviral containing shRNA targeting Runx2 (lentiviral-Runx2 shRNA) or control shRNA was designed and synthesized with Genscript (Nanjing, China). Cells were planted in a 6-well plate, followed by adding the lentiviral-Runx2 shRNA or control shRNA and being incubated for 48 h. The transfection was mediated by the reagent lipofectamine 2000 (Invitrogen, California, USA). The knockdown efficacy was determined using the Western blotting assay.

### 3-(4,5)-dimethylthiahiazo (-z-y1)-3,5-di- phenytetrazoliumromide (MTT) assay

After different treatment strategies, cells were treated with 0.25 mg/ml MTT (Sigma, Missouri, USA) at 37°C for 3 h, followed by removing the medium and adding the dimethyl sulfoxide to produce blue formazan. Then, the microplate reader (Mindray, Shenzhen, China) was used to measure the absorbance at 630 nm [17].

### Lactate dehydrogenase (LDH)

fter necessary treatment, 50 μL of the cell culture medium was collected from each well and mixed with an equal amount of 50 µL reaction buffer. OD value was recorded at 450 nm to index the amount of LDH.

### ALP activity assay

Cells were seeded in a 6-well plate and cultured in the osteogenic medium. The activity of ALP was measured using the ALP activity kit (Sigma-Aldrich, USA). Briefly, after necessary treatment, cells were lysed and the protein concentration was assessed using a BCA kit. Samples were mixed with reaction buffer (0.1 M 2-amino-2-methyl-1-propanol, 1 mM MgCl_2_, 8 mM p-nitrophenyl phosphate disodium) and incubated at 37°C for 4 minutes. The reaction was stopped with 0.1 N NaOH. Absorbance at 405 nm was used to index ALP activity.

### mRNA isolation and real-time PCR

The Trizol reagents were utilized to extract the cellular total RNA from cells and the concentration of RNA was measured by detecting the optical density at 260 nm, followed by being transcribed into cDNA utilizing the PrimeScript RT Master Mix Kit (Takara, Tokyo, Japan). The Sybr Premix Ex Taq Kit (Takara, Tokyo, Japan) was used to perform the RT-PCR in the present study. The expression of genes was determined with the 2^−ΔΔCt^ method after normalization to GAPDH. The primer sequences used are listed as follows: ALP (Forward: 5ʹ-GGGGACATGCAGTATGAATT-3ʹ, Reverse: 5ʹ- GGCCTGGTAG TTGTTGTGAG −3ʹ); Col1α1 (Forward: 5ʹ- GCAACAGTCGCTTCACCTACA-3ʹ, Reverse: 5ʹ- CAATGTCCAAGGGAGCCACAT-3ʹ); Ocn (Forward: 5ʹ-TGCTTG TGACGAGCTATCAG-3ʹ, Reverse: 5ʹ-GAGGACAGGGAGGATCAAGT-3ʹ); BMP-2 (Forward: 5ʹ-AACACCGTGCGCAGCTTCCATC-3ʹ, Reverse: 5ʹ-CGGA AGATCTGGAGTTCTGCAG-3ʹ); RUNX-2 (Forward: 5ʹ-CTTCATTCGCCTCACA AAC-3ʹ, Reverse: 5ʹ-GTCACTGCGCTGAAGA-3ʹ); Col1a1 (Forward: 5ʹ- GCAACAGTCGCTTCACCTACA-3ʹ, Reverse: 5ʹ-CAATGTCCAAGGGAGCCAC AT-3ʹ); Col1a2 (Forward: 5ʹ-CCCAGAGTGGAACAGCGATT-3ʹ, Reverse: 5ʹ- ATGAGTTCTTCGCTGGGGTG-3ʹ); Sp7 (Forward: 5ʹ-GCAGGCATCCACGC AGGCATCTC-3ʹ, Reverse: 5ʹ-CCTGGCCCTGACCACCACCTAGC-3ʹ); Fgfr2 (Forward: 5ʹ-GCTATAAGGTACGAAACCAGCAC-3ʹ, Reverse: 5ʹ-GGTTGATGG ACCCGTATTCATTC-3ʹ); Fgfr3 (Forward: 5ʹ-GCCTGCGTGCTAGTGTTCT-3ʹ, Reverse: 5ʹ-CCTGTACCATCCTTAGCCCAG-3ʹ); GAPDH (Forward: 5ʹ-AGGTCG GTGTGAACGGATTTG-3ʹ, Reverse: 5ʹ- TGTAGACCATGTAGTTGAGGTCA-3ʹ).

### Alizarin red S staining assay

Cells were planted in a 6-well plate, followed by being treated with different strategies for 28 days. Then, 4% paraformaldehyde was added to fix cells, followed by adding 2% Alizarin Red S (Sigma, Missouri, USA) to examine the extracellular matrix calcification for 15 min. Lastly, 400 μl 10% (w/v) cetylpyridinium chloride (Sigma, Missouri, USA) and 10 mM sodium phosphate solution were added into the wells. Lastly, the microplate reader (BMG LABTECH, Offenburg, Germany) was utilized to detect the absorbance at 562 nm [18].

### Protein isolation and western blotting analysis

After isolating total proteins from cells with the lysis buffer, a BCA kit was used to quantify the isolated proteins, and approximately 30 μg samples were added, followed by separation with 10% SDS-PAGE. Then the protein was transferred to the PVDF membrane (Millipore, Massachusetts, USA) and then incubated with 5% skim milk. The membrane was then immersed in the primary antibodies against BMP-2 (1:2000, Proteintech, China), Ocn (1:2000, Proteintech, China), ALP (1:1000, Proteintech, China), Runx2 (1:2000, Proteintech, China), Sp7 (1:2000, Proteintech, China), Fgfr2 (1:2000, Proteintech, China), Fgfr3 (1:3000, Proteintech, China), p-p38 (1:1000, Proteintech, China), p38 (1:3000, Proteintech, China), p-Akt (1:1000, Proteintech, China), Akt (1:3000, Proteintech, China), and β-actin (1:8000, Proteintech, China). Subsequently, the membrane was incubated with the secondary antibody (1:2000, Proteintech, China) at room temperature for 1.5 h. Lastly, the bands were visualized using ECL solution, followed by quantification with the Image J software.

### Statistical analysis

Data were analyzed using the GraphPad Prism version 8.3.0 and were presented as mean ± standard deviation (S.D.). T-test was used to compared two independent data and the data among groups were compared using the two-way analysis of variance (ANOVA) method, while p < 0.05 was taken as a significant difference.

## Results

Firstly, the cytotoxicity of Omarigliptin in MC3T3‑E1 cells was measured to screen the optimal concentration. Secondly, the effects of Omarigliptin on ALP activity, mineralization, and the expressions of osteoblastic differentiation biomarkers such as BMP-2, Ocn, and ALP genes in MC3T3-E1 cells were assessed. Lastly, we proved that the beneficial effects of Omarigliptin were mediated by the activation of p38, Akt, and Runx2.

### The effect of Omarigliptin on cell viability of MC3T3‑E1 cells

**Figure 1.**
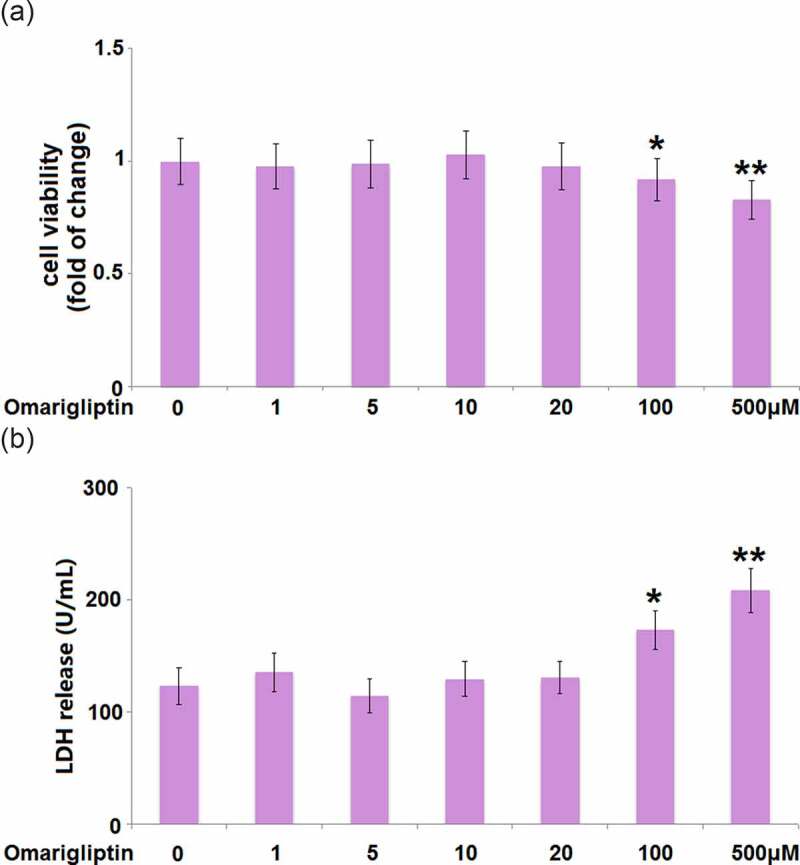
The effect of Omarigliptin on cell viability of MC3T3‑E1 cells. Cells were treated with Omarigliptin at varying concentrations (0, 1, 5, 10, 20, 100, 500 μM) for 14 days, (a) the cell viability and (b) LDH release were determined (*, **, P < 0.05, 0.01 vs. vehicle group, n = 5–6)

To determine the optimized concentration of Omarigliptin to be used for the incubation with MC3T3‑E1 cells, the cells were treated with Omarigliptin at various concentrations (0, 1, 5, 10, 20, 100, 500 μM) for 14 days, followed by evaluating the cell viability using the MTT assay. We found that when the concentration was lower than 20 μM (Figure 1a), the cell viability remained around 100%. However, when the concentration of Omarigliptin was higher than 100 μM, significantly decreased cell viability was observed. Consistently, the LDH release assay demonstrates that Omarigliptin with lower concentrations (less than 20 μM) did not increase the release of LDH. In contrast, exposure to 100 and 500 μM Omarigliptin significantly stimulated the release of LDH (Figure 1b). Therefore, in the subsequent experiments, 20 μM was used as the incubation concentration of Omarigliptin.

### The impact of Omarigliptin on ALP activity and mineralization in MC3T3‑E1 cells

**Figure 2.**
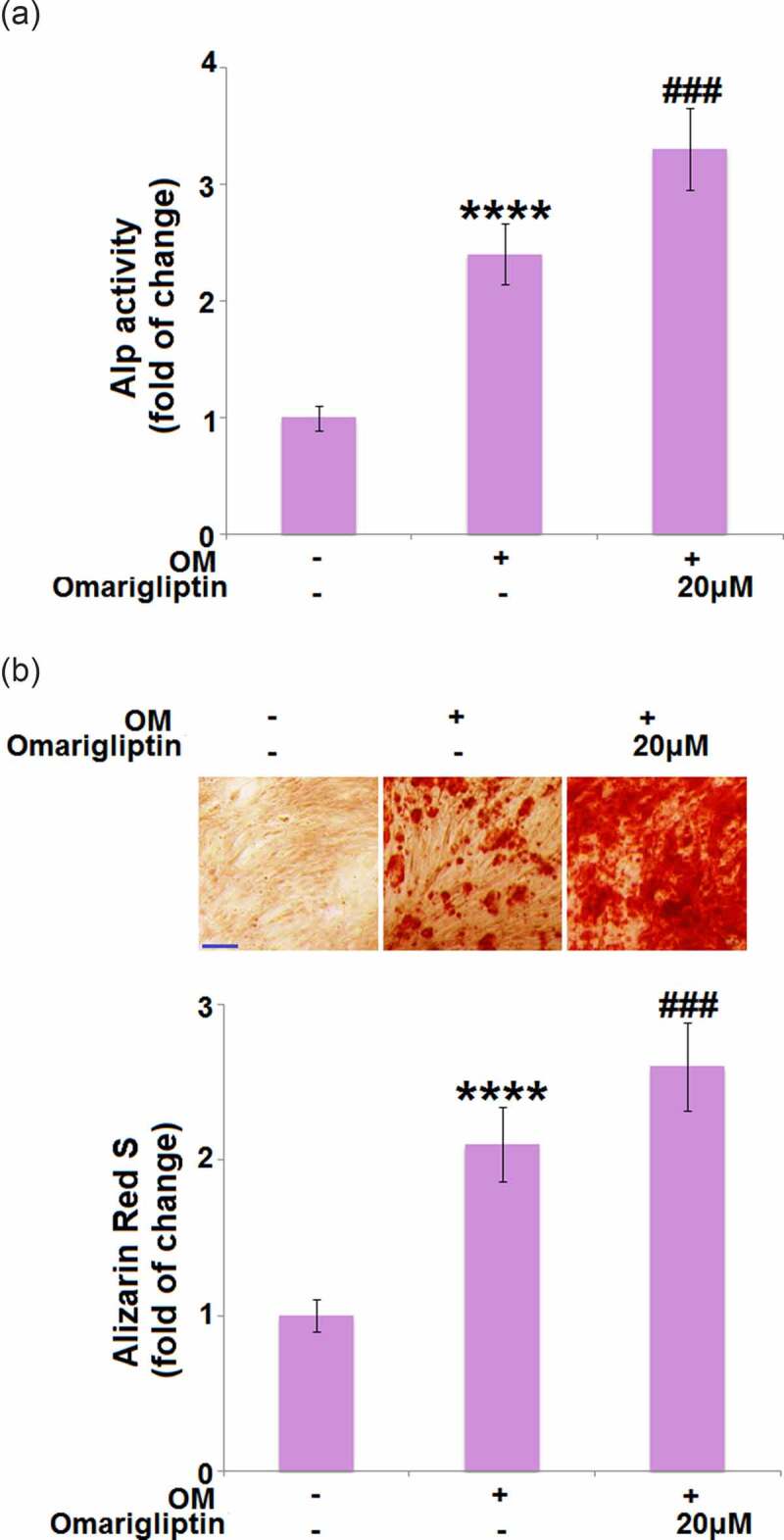
The effect of Omarigliptin on ALP activity and mineralization in MC3T3‑E1 cells. Cells were cultured with osteogenic medium (OM) and Omarigliptin (20 μM) for 14 days. (a) The Alp activity; (b) Alizarin Red S staining assay in MC3T3-E1 cells. Scale bar, 200 μm (****, P < 0.0001 vs. vehicle group; ###, P < 0.001 vs. OM treatment group, n = 6)

ALP activity and mineralization are critical signs for osteoblastic differentiation [19]. Cells were cultured with osteogenic medium with or without Omarigliptin (20 μM) for 14 days, followed by evaluating the ALP activity and mineralization. Compared to the MC3T3‑E1 cells, the ALP activity (Figure 2a) and mineralization (Figure 2b) were significantly enhanced by the osteogenic medium, then further elevated by 20 μM Omarigliptin, indicating a potential inhibitory effect of Omarigliptin on osteoblastic differentiation.

### The effect of Omarigliptin on the expression of BMP-2, Ocn, and ALP in MC3T3‑E1 cells

**Figure 3.**
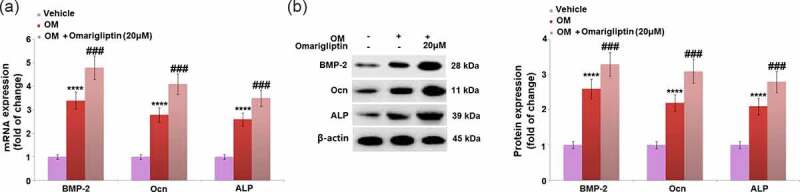
The effect of Omarigliptin on the expressions of BMP-2, Ocn, and ALP in MC3T3‑E1 cells. The cells were cultured with osteogenic medium (OM) and Omarigliptin (20 μM). (a) The mRNA BMP-2, Ocn, and ALP; (b) The Protein expression level of BMP-2, Ocn, and ALP (****, P < 0.0001 vs. vehicle group; ###, P < 0.001 vs. OM treatment group, n = 5–6)

We further measured the expression levels of important factors involved in the regulation of osteoblastic differentiation, such as BMP-2 [20], Ocn [21], and ALP [22]. We found that BMP-2, Ocn, and ALP (Figure 3a-b) were significantly upregulated in osteogenic medium-cultured MC3T3‑E1 cells at both the mRNA and protein levels, and pronouncedly further upregulated by 20 μM Omarigliptin, revealing the regulatory function of Omarigliptin on osteoblastic differentiation is associated with these factors.

### *The effect of Omarigliptin on the expressions of* Col1a1 *and* Col1a2 *in MC3T3‑E1 cells*

**Figure 4.**
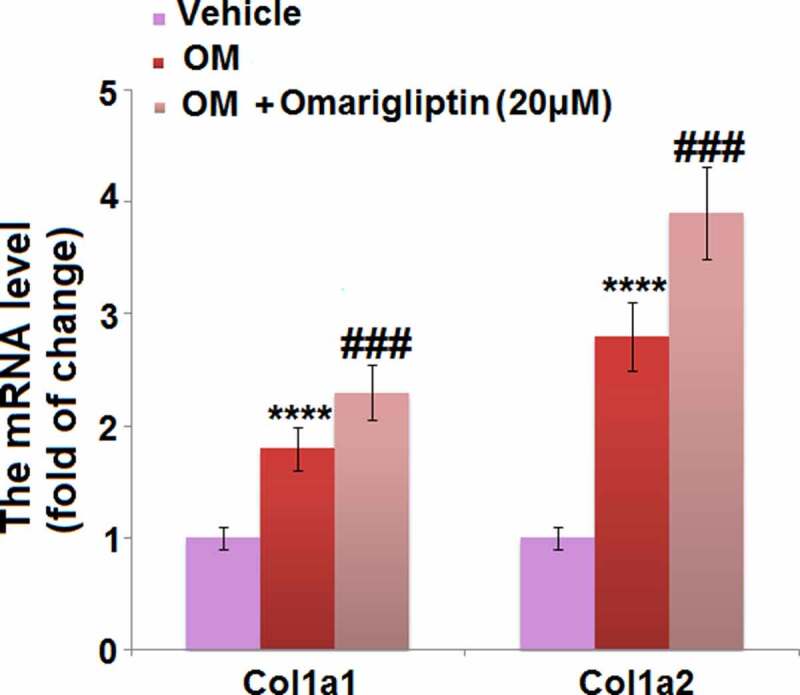
The effect of Omarigliptin on the expressions of Col1a1 and Col1a2 in MC3T3‑E1 cells. The cells were cultured with osteogenic medium (OM) and Omarigliptin (20 μM). The mRNA level of Col1a1 and Col1a2 (****, P < 0.0001 vs. vehicle group; ###, P < 0.001 vs. OM treatment group, n = 5–6)

Collagens are the main components of the extracellular matrix of bone tissues and are encoded for by *Col1a1* and *Col1a2* [23]. We found that the elevated expression levels of *Col1a1* and *Col1a2* in osteogenic medium-cultured MC3T3‑E1 cells were further promoted by 20 μM Omarigliptin (Figure 4), indicating a potential protective effect of Omarigliptin against extracellular matrix degradation.

### The effect of Omarigliptin on Runx-2 and SP7 in MC3T3‑E1 cells

**Figure 5.**
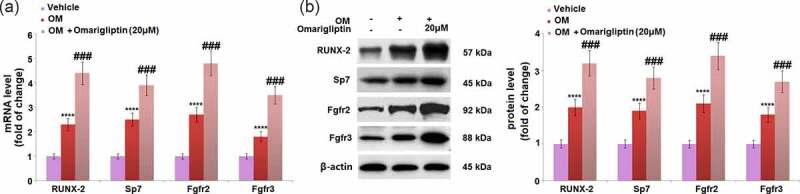
The effect of Omarigliptin on the expressions of RUNX-2, SP7, Fgfr2, and Fgfr3 in MC3T3‑E1 cells. The cells were cultured with osteogenic medium (OM) and Omarigliptin (20 μM). (a) The mRNA expression levels of RUNX-2, Sp7, Fgfr2, and Fgfr3; (b) The protein level of RUNX-2, Sp7, Fgfr2, and Fgfr3 (****, P < 0.0001 vs. vehicle group; ###, P < 0.001 vs. OM treatment group, n = 5–6)

The Runx2/SP7 pathway is an important pathway for the transcriptional factors of osteoblastic differentiation [24]. The expression levels of key proteins in the Runx2/SP7 pathway were determined. We found that compared to MC3T3‑E1 cells cultured in the α-MEM medium, the gene (Figure 5a) and protein (Figure 5b) expression levels of Runx-2, Sp7, Fgfr2, and Fgfr3 were significantly elevated in MC3T3‑E1 cells cultured in osteogenic medium, which was dramatically further reinforced by 20 μM Omarigliptin, indicating an activation effect of Omarigliptin on the Runx2/SP7 pathway.

### The MAP kinase p38 and Akt signaling pathways are involved in Omarigliptin-induced cell differentiation in MC3T3‑E1 cells

**Figure 6.**
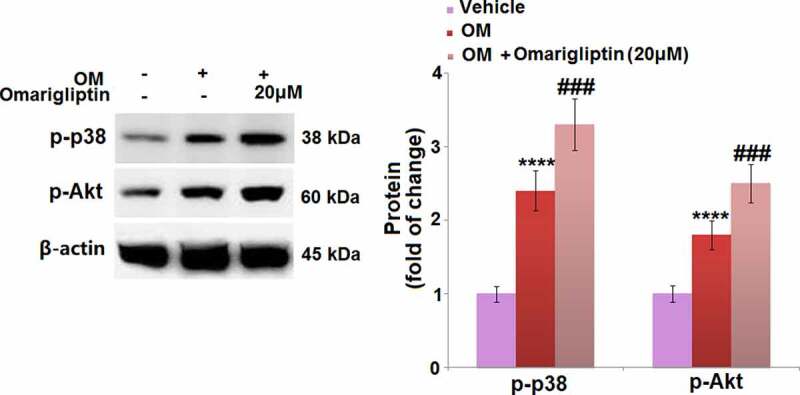
The MAP kinase p38 and Akt signaling pathways were involved in Omarigliptin-induced osteogenic differentiation in MC3T3‑E1 cells. The cells were cultured with osteogenic medium (OM) and Omarigliptin (20 μM). Western blots of p-p38 and p-Akt (****, P < 0.0001 vs. vehicle group; ###, P < 0.001 vs. OM treatment group, n = 5–6)

To further investigate the mechanism underlying the Omarigliptin-induced cell differentiation in MC3T3‑E1 cells, the activities of the MAP kinase p38 and Akt signaling pathways were determined. As shown in Figure 6, the upregulated p-p38 and p-Akt in the osteogenic medium-cultured MC3T3‑E1 cells were significantly further upregulated by 20 μM Omarigliptin, indicating an activation effect of Omarigliptin on the MAP kinase p38 and Akt pathways.

### Runx-2 was involved in Omarigliptin-induced osteoblastic differentiation

**Figure 7.**
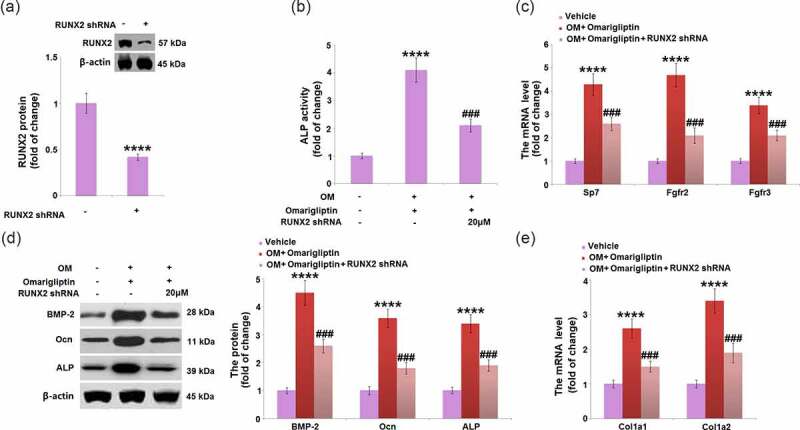
RunX2 was involved in Omarigliptin-induced osteoblastic differentiation in MC3T3‑E1 cells. The cells were infected by lentiviral-RUNX2 shRNA or control shRNA and then treated with osteogenic medium (OM) and 20 μM Omarigliptin. (a). RUNX2 protein level was determined; (b) The Alp activity; (c) The mRNA level of Sp7, Fgfr2, and Fgfr3; (d) The mRNA level of BMP-2, Ocn, ALP; (e) The mRNA level of Col1a1 and Col1a2 (****, P < 0.0001 vs. vehicle group; ###, P < 0.001 vs. OM+ Omarigliptin group, n = 5–6)

To investigate the key mediator involved in the Omarigliptin-induced cell differentiation, cells were infected by lentiviral- Runx2 shRNA or control shRNA for 48 h and then treated with 20 μM Omarigliptin. Firstly, the knockdown of Runx 2 was confirmed by the Western blotting assay (Figure 7a). In addition, compared to the osteogenic medium-cultured cells, ALP activity was significantly elevated by Omarigliptin but greatly suppressed by the transfection with Runx2 shRNA (Figure 7b). The upregulated Sp7, Fgfr2, Fgfr3, BMP-2, Ocn, and ALP (Figure 7c-d) in Omarigliptin- treated cells cultured in the osteogenic medium were greatly reversed by the transfection with Runx2 shRNA. Lastly, the increased expression levels of *Col1a1* and *Col1a2* in the Omarigliptin group were dramatically repressed by the knockdown of Runx2 in osteogenic medium-cultured MC3T3‑E1 cells (Figure 7e). These data collectively reveal that Omarigliptin-induced osteoblastic cell differentiation was significantly abolished by the knockdown of Runx2.

## Discussion

The BMP pathway not only regulates osteoblastic differentiation but also impacts the production of new bone [25]. BMP-2 is an important subunit of the pathway, and it is reported that it triggers the differentiation from mesenchymal stem cells into mature osteoblasts, which further differentiate into osteocytes. BMP-2 is publicly recognized as the most powerful growth factor that is used for inducing ossification [25]. During the progression of osteoblastic differentiation, the expression levels of osteoblasts specific transcriptional factors, such as Runx2 and Osx, are significantly elevated, further enhancing the differentiation of osteoblasts. As the downstream proteins in the BMP-2 pathway, Runx2 plays a vital role in the stage of the directional differentiation of mesenchymal stem cells while Osx exerts its function in the advanced stage [26]. In addition, it is reported that other osteoblastic biomarkers, such as ALP, MEPE, Col-I, Ocn, Opn, and BSP, could be significantly upregulated by the BMP signaling pathway to facilitate bone formation [27]. We found that osteoblastic differentiation was greatly induced by Omarigliptin based on osteogenic medium incubation, evidenced by the results of measurements on ALP activity and mineralization. In osteogenic medium-cultured MC3T3‑E1 cells, we found that BMP-2, as well as other osteoblastic biomarkers, such as Ocn, Col-I, and ALP, were significantly upregulated, further verifying osteoblastic differentiation induced by osteogenic medium incubation. After treatment with Omarigliptin, the expression levels of BMP-2 and the osteoblastic biomarkers were significantly further elevated, which was consistent with the results of ALP activity and mineralization. These data reveal that the Omarigliptin facilitated the BMP-2 mediated osteoblastic differentiation.

Under the stimulation of hormones, such as epinephrine and parathyroid hormone, the p38 MAPK pathway is reported to exert a critical effect in facilitating osteoblastic differentiation by enhancing the expression levels of multiple bone factors [14,28]. The PI3K/Akt pathway is a classic signaling pathway involved in multiple types of cellular biological processing, such as proliferation and apoptosis, which is reported to be a positive regulatory pathway in the progression of osteoblastic differentiation [29]. Our results indicate the p38 and Akt pathways were both activated by Omarigliptin, which might be associated with Omarigliptin-induced osteoblastic cell differentiation. In later investigations, the effects of Omarigliptin on more key proteins in these two pathways, such as MAPK, PI3K, and ERK1/2, will be further explored to verify its regulatory property on the p38MAPK and PI3K/Akt pathways.

The directional differentiation of mesenchymal stem cells or pre-osteoblastic cells into osteoblasts can be facilitated by Runx2 [30]. *In vitro* experiments have indicated that in osteoblasts and osteoclasts cultural media, receptor activator of nuclear factor-kappa B ligand (RANKL) could be dramatically upregulated in osteoblasts and mesenchymal stem cells by the overexpression of Runx2, indicating Runx2 significantly facilitated osteoblast differentiation and function [31]. Recently, Runx2 and Sp7 have been reported to be necessary transcription factors for osteoblast differentiation by regulating Fgfr2 and Fgfr3 [32]. Our data reveal that Runx2 in osteogenic medium-cultured MC3T3‑E1 cells was greatly upregulated, accompanied by the elevated expression levels of Sp7, Fgfr2, and Fgfr3, which further verified the osteoblast differentiation. After the treatment with Omarigliptin, Runx2, Sp7, Fgfr2, and Fgfr3 were further upregulated, indicating Omarigliptin facilitated the progression of osteoblast differentiation in osteogenic medium-cultured MC3T3‑E1 cells. Lastly, Omarigliptin-induced osteoblastic cell differentiation was significantly abolished by the knockdown of Runx2, revealing that Runx2 might be an important mediator for Omarigliptin- induced osteoblastic differentiation. As osteoporosis is a common age-related disease, many studies have focused on therapeutic strategies for its treatment. Fu Y et al. demonstrated that the LncRNA ROR/miR-145-5p axis modulates osteoblasts proliferation and apoptosis in osteoporosis [33]. In future work, the therapeutic function of Omarigliptin against Osteoporosis will be researched by treating the Osteoporosis animal model using the applicable doses.

## Conclusion

Overall, our study identifies a previously unknown pharmacological function of Omarigliptin in promoting osteoblastic differentiation by increasing ALP activity, stimulating mineralization, and enhancing the expressions of the relevant biomarker genes. Additionally, our work on the p38MAPK, Akt, and Runx2 pathways as important modulators for this role of Omarigliptin provides novel insights into the molecular mechanism. These findings suggest that Omarigliptin may be a novel therapeutic agent for Osteoporosis.

## Data Availability

Requests for data and materials should be addressed to the corresponding author.
